# Pathways between climate change and HIV health in rural Kenya: a qualitative analysis

**DOI:** 10.1038/s41598-026-52085-7

**Published:** 2026-05-08

**Authors:** Tammy M. Nicastro, Gladys Odhiambo, Stanley Jawuoro, Elly Weke, Elizabeth A. Bukusi, Yihui A. Yang, Helen A. Harris-Fry, Suneetha Kadiyala, Sheri D. Weiser

**Affiliations:** 1 Department of Population Health, London School of Hygiene & Tropical Medicine, Keppel Street, London, WC1E 7HT UK; 2 Department of Obstetrics, Gynecology, and Reproductive Sciences, University of California, San Francisco, San Francisco, CA USA; 3 Kenya Medical Research Institute, Nairobi, Kenya; 4 Geisel School of Medicine, Dartmouth College, Hanover, NH USA; 5 Department of Medicine, University of California, San Francisco, San Francisco, CA USA

**Keywords:** Climate change, Agriculture, HIV, Kenya, Climate sciences, Health care

## Abstract

Climate change-related severe weather events are heavily impacting regions with the highest prevalence of HIV, creating additional vulnerabilities for already vulnerable populations. Little is understood about how people perceive or experience the mechanisms by which extreme weather events affect the health of people living with HIV (PLHIV). We conducted a qualitative study using in-depth, semi-structured interviews with 40 PLHIV enrolled in a cluster randomized clinical trial that included 8 pairs of health facilities in rural Western Kenya, who were 18 years or older, receiving ART for > 6 months; had moderate to severe food insecurity; and practiced smallholder farming with access to surface water or aquifers. Intervention participants received a loan to purchase an irrigation pump and farming inputs and were provided climate-responsive, sustainable agriculture and financial literacy training. This study did not evaluate the impacts of the clinical trial. We aimed to understand participant perceptions of how climate change impacted their HIV health, and associated pathways for these impacts. Interviews were transcribed, translated, and double coded using an inductive-deductive-abductive thematic content approach. Almost all participants noted that droughts, flooding, and elevated temperatures had serious negative impacts on their health and wellbeing. Extreme weather negatively impacted their health via five key pathways, with the first being most prominent: (1) decreases in agricultural yields and income; (2) increased food insecurity and undernutrition; (3) medication non-adherence, missed clinic visits, and infrastructure erosion; (4) increased infections, and (5) displacement and forced migration. Several pathways were interrelated, with decreased agricultural yields and income being proximal to most other pathways. Participants perceived pathways by which severe weather negatively impacted their HIV health, and these pathways were bi-directional and reinforcing. Food and livelihood sources were devastated, and housing and infrastructure were negatively affected, causing a cascade of nutrition and health vulnerabilities. Understanding the contexts in which PLHIV are vulnerable to impacts of climate change will be essential to establish climate-responsive policies that can interrupt the pathways identified here. Cross-sector collaboration between the Kenyan Ministries of Agriculture and Health to develop climate-responsive policies to support PLHIV should be prioritized. The clinical trial start date was June 23, 2016. Trial Registration: Registered with ClinicalTrials.gov Identifier: NCT02815579.

**Supplementary Information:**

The online version contains supplementary material available at 10.1038/s41598-026-52085-7.

## Introduction

Climate change poses a serious public health concern^1,2^. Earlier studies show that it affects health through several exposure pathways, including extreme heat, poor air quality, reduced food and water quality, changes in infectious disease dynamics, disruption of services, and population displacement^2–4^. These pathways contribute to a wide range of adverse health outcomes, including heat-related mortality, adverse childbirth outcomes, infectious diseases, mental health conditions, and food and nutrition insecurity^5–7^.

These health impacts are often greater among vulnerable populations with higher exposure to climate hazards and limited capacity to adapt^8,9^. Individuals with existing health conditions, including people living with HIV (PLHIV), older adults, and children, may be particularly susceptible to the effects of climate change, especially where financial, material, and social resources to respond are constrained^8,12–16^.

Climate change impacts on HIV health outcomes, including HIV progression, immune function, opportunistic infections, treatment response, or comorbidities have been notably overlooked^2^. Sub-Saharan Africa (SSA) is one of the regions of the world most affected by climate change and also has the highest prevalence of HIV and people living in poverty, creating a vulnerability nexus requiring attention^10^. Understanding the pathways through which PLHIV may experience negative impacts from climate change is important to support already vulnerable people from additional threats to their health and well-being.

One way that climate change may impact HIV health is through nutrition insecurity which can lead to compromised immunity^10^. Extreme or unpredictable weather, such as higher temperatures, sporadic and unpredictable rainfall, floods, and more frequent and more severe droughts, can reduce agricultural yields and increase food prices, reducing food security and dietary quality of affected populations^11,12^. A large body of modeling studies have predicted negative impacts of climate change on agricultural productivity and health^13–17^. As a result, smallholder farmers dependent upon subsistence food production will be at greater risk of food insecurity, reduced income, poor diet quality, and the negative downstream health effects of both. Projections from Kenya, where over a third of the population is already food insecure, indicate that by 2050 an additional 3.2 million people will be at risk of hunger due to climate change-related rising temperatures and increased floods and droughts^18^.

Climate change poses a particularly serious threat to the food security of smallholder farmers living with HIV. This is due to constraints in their coping mechanisms to address climate-related agricultural shocks, as households often struggle to meet farming labor demands due to illness and higher healthcare expenditures^19,20^. In turn, food insecurity and poor nutrition can negatively affect health outcomes of PLHIV through reinforcing and bidirectional mechanisms^21–31^. PLHIV taking antiretroviral therapy (ART) and those suffering from lipodystrophy are estimated to have a 10% and 19% (respectively) higher resting energy expenditure^32^, while PLHIV suffering an opportunistic infection have a 10–30% increased resting energy expenditure, as compared to healthy populations^33^. As such, PLHIV have increased energy requirements, and failure to meet energy needs can put PLHIV at risk of wasting, progression to AIDS, and increased morbidity and mortality. Specifically, poor nutrition, even among PLHIV taking ART, compromises immune function which can lead to opportunistic infections. In Kenya, HIV is a public health crisis, with 1.5 million PLHIV, 70% of whom are food insecure and deficient in micro- and macro-nutrients^34–37^.

Another hypothesized mechanism through which climate change could pose threats to PLHIV is excessive flooding, which causes destruction of roads and health facilities, making it difficult to reach health clinics or agricultural markets^10^. A higher prevalence of infectious diseases such as malaria and diarrhea from excess standing water and contaminated water systems has been associated with floods and stagnant water^38^. These mechanisms could potentially put PLHIV at higher risk of opportunistic infections^39^, at the same time potentially affecting access to health care^40^ and affordable food^41^. Yet little data exists on the mechanisms through which climate change affects HIV health^13,42^.

Our study provides a unique contribution to the literature by being one of the first to examine PLHIV’s experiential pathways between climate change and HIV health, using qualitative methods to understand participants’ experiences of weather shocks, and key protective and risk factors for HIV health^43,44^. To frame this inquiry, we draw on Lieber et al.’s^10^ conceptual framework linking climate change to HIV health outcomes through pathways of food insecurity, infectious diseases, forced migration, and erosion of infrastructure^10^. Our analysis utilizes critical realist theory^45,46^ which, when applied to our study, posits that individuals will respond differently to the same initial exposure to climate change, even when they are within the same social system, based on their individual context and capacity to adapt^47^. By applying critical realist theory, we can better understand the highly complex processes by which participants respond to climate change and identify which experiences of climate change relate to an individual’s particular context, and which are shared experiences that are common to all participants^48,49^.

## Methods

### Research design and setting

This qualitative inquiry is a sub-study that uses qualitative data from a large, cluster-randomized controlled trial (CRCT), *the ‘Shamba Maisha’* trial^50^, which evaluated the impact of a multicomponent agricultural intervention targeted to PLHIV on their HIV clinical outcomes.

### Shamba Maisha trial

The 24-month *Shamba Maisha* intervention consisted of a bank loan to purchase an irrigation pump, seeds, pesticides, and fertilizers, and sustainable farming and financial management training. The study was conducted in Western Kenya’s Nyanza Region, which has the highest prevalence of HIV (10%)^51^ in Kenya, where 40–80% live in poverty, and almost all PLHIV experience food insecurity^21,52,53^.

Around 75% of rural Kenyans are employed in the agriculture sector, 70% are smallholder farmers, and 40% nationally earn an income from agriculture^54,55^. The agriculture sector is responsible for 80% of the population’s employment, income, and food security collectively^55^, and smallholders supply the majority of Kenya’s food^54,56,57^, making the stability of agricultural yields critically important to Kenya’s public health.

People in this region are primarily engaged in subsistence agriculture (70%)^58^. People grow a mixture of crops such as grains, fruit, and vegetables for home consumption, with the surplus sold in the local markets^59^. In this decade, Nyanza Region has seen more severe and frequent flooding, unpredictable rain, longer droughts, and rising temperatures as compared to the previous decade, with trends showing increased vulnerability^60,61^.

Between June 2016 and December 2017, Shamba Maisha participants were enrolled from 16 health facilities in Nyanza Region that were randomly assigned to either the control or intervention arm of the *Shamba Maisha* trial^50^. Patients attending these health facilities were eligible to participate in the trial if they were: on ART for ≥ 6 months, ≥ 18 years of age, moderately to severely food insecure or BMI < 18.5 kg/m^2^ at study enrollment and had access to farming land that could be ploughed with relatively flat topography and water for use with an irrigation pump.

### Study population and recruitment for qualitative sub-study

We leveraged the *Shamba Maisha* study^50^ to collect data for the current sub-study, the aim of which was to understand how PLHIV experience climate change, and the pathways to food security, nutrition, and HIV health outcomes. At baseline, sociodemographic factors were similar among both intervention and control arms (see Table 4). HIV viral load detection was 5% (viral load detection limit, ≤ 1000 copies/ml). We sampled from 10 facilities in communities that experienced extreme flooding and/or prolonged droughts during the previous year, which included 14 of the 16 enrolled sites at the time of our recruitment. Inclusion criteria were: (1) participants who were actively farming and (2) attending study activities. *Shamba Maisha* study research assistants and study managers helped to identify participants who met these criteria using their knowledge of participants and data from the baseline survey from the *Shamba Maisha* trial to ensure distribution across age and geography. As the goal of this sub-study was to examine PLHIVs’ lived experiences of climate change, and not estimation of *Shamba Maisha’s* impacts, we purposively chose men and women from both intervention and control communities, with the groups evenly distributed by gender and intervention arm. We included an equal number of control participants that were not influenced by the intervention to account for possible early intervention benefits for those enrolled in the intervention arm. Two field researchers screened participants for inclusion in the climate change sub-study through phone calls, during which the researchers described the time commitment for the study, details of interview topics, and determined willingness to participate. No other methods were used to recruit participants. To capture variation in lived experience and reach data saturation in the primary pathways^62^, we planned to conduct interviews with a minimum of 40 participants equally divided between men and women. All participants who were approached agreed to participate, and all completed the study.

### Data collection

We conducted in-depth semi-structured interviews and followed a topic guide that covered domains of interest modeled after the pathways in Lieber et al.^10^. These included participants’ perceptions and experiences of the impacts of climate change on agriculture, food security and diet quality, income, general health, and HIV health. TMN led the interview guide development which included four other researchers and had extensive local input (SDW, EW, GO, SJ) and underwent multiple iterations.

Interviews were conducted by two local research assistants (one female and one male) fluent in local languages (Dholuo and Kiswahili) and English. Interviewers were hired to conduct qualitative interviews only and were not involved with other aspects of the intervention. The interviewers held at least a bachelor’s degree, and both had extensive training conducting qualitative interviews such as using exploratory, non-leading, non-judgmental questions and probing for participants’ detailed experiences. Interviewers also had extensive experience in the study’s topic areas. TMN conducted a 2-day in-person training course for the interviewers that included a presentation by the *Shamba Maisha* study manager to explain key features of the study activities and sites and a presentation by the data manager to review our protocol for storing physical and digital copies of interview audio recordings, interview memos, and transcripts. The training included presentations and group exercises on semi-structured interview principles, coding techniques, confidentiality, and sensitivity training on interview topics we anticipated may be uncomfortable for some participants. These group exercises included the interviewers, a researcher leading the maternal nutrition qualitative sub-study (AMD), and TMN.

We piloted the interview guide, with two participants from the main *Shamba Maisha* trial who were not part of this qualitative study cohort, to ensure consistent interviewers’ approach and then refined the questions to reduce repetition and improve the question sequence. TMN led this review process in-person with the interviewers and discussed changes with SDW by phone.

One in-depth semi-structured interview was conducted and recorded with each participant between September 2018 and January 2019 with interviewers gender-matched to participants. All interviews were conducted in a safe and confidential environment either at the participant’s home, farm, or at the clinic in a private area with only the interviewer and the participant present. Participants provided written informed consent. Interviewers had no previous contact with the participants. Participants were compensated 400 Kenyan shillings (KSH) (equivalent to 4 US$) for their travel expenses (if conducted at a clinic) and 400 KSH for their time.

The interview guide was further refined after the initial interviews were conducted. TMN led a process with the interviewers and NB (a trainee mentored by SDW) to remove questions that gave repetitive responses and re-order questions to match the natural sequence of how participants discussed climate change impacts. All revisions were reviewed by SDW and findings from all interviews (except the two pilot interviews described above) were included in the analysis and reported.

### Ethics statement

Ethics approval for this study was obtained from the Kenya Medical Research Institute (KEMR1/SERU/CMR/3027), and the University of California, San Francisco (IRB # 14-15592). The clinical trial is registered at ClinicalTrials.gov (NCT02815579). All methods were carried out in accordance with relevant guidelines and regulations. All procedures involving human participants were performed in accordance with the ethical standards of the KEMRI and UCSF research committees and with the Declaration of Helsinki and its later amendments or comparable ethical standards. Transcripts were pseudo anonymized by removing participant names and replacing them with a unique participant number that could only be traced back to the participant through a document stored in a double password-protected file. Participant responses were redacted if they named places or people that would identify them.

### Data analysis

Interviewers led the transcription process for their interviews by transcribing then translating verbatim using emic words and phrases to retain local meaning that otherwise would not be translated accurately. TMN then reviewed the transcripts with the interviewers to ensure translation accuracy and any questions discussed with the interviewer with support from SDW and NB. Fieldnotes were attached to each transcript, and the location, timepoint, and gender of the participants were included.

Interview transcripts were uploaded into Dedoose web application for managing, analyzing, and presenting qualitative research data (Version 9.0, Los Angeles, California). The codebook development and coding were led by TMN, with support from SDW and NB. An initial codebook was established based on topics covered in the interview guide and covering pathways described in Lieber et al.’s framework^10^. Two transcripts were double coded by TMN and NB using thematic content analysis to deductively code data supporting a priori themes and theories or inductively code new themes that did not correspond to any pre-existing assumptions^63^. More detailed and contextual data was coded as a sub-theme under an existing broad code. TMN and NB then reviewed each other’s coded excerpts, discussed any discrepancies, decided how codes in question would be consistently used, and wrote a memo explaining the discrepancy and resolution. TMN then double coded the remaining transcripts with the interviewers using the process described and whereby the interviewers coded the interviews they conducted. Data saturation was reached at approximately 14 transcripts for men and women participants (28 total transcripts); however all transcripts were coded to clarify themes, add specificity to findings, and confirm the strength of identified themes. Additionally, we used inductive reasoning to interrogate the data for contexts associated with outcomes and coded accordingly. This allowed us to develop new theories of mediating pathways after comparing multiple datapoints to test and confirm our hypotheses^64,65^.

**Fig. 1:**
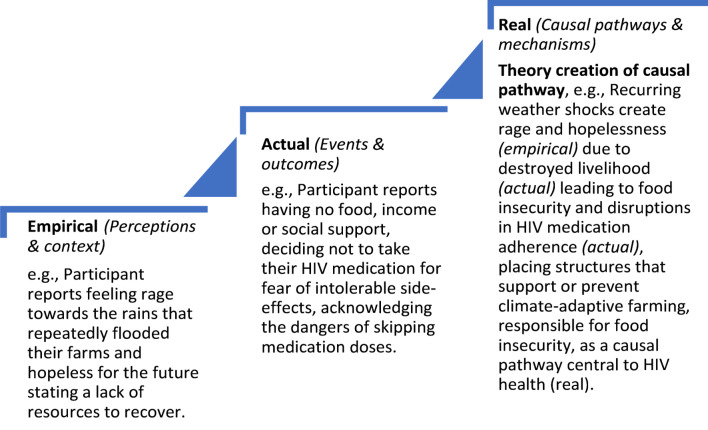
Data analysis with illustrative example using critical realist theory.

Critical realist theory was used to guide the analysis. Critical realism supports the context-mechanism-outcome configuration used to answer the question ‘What works for whom and under what circumstances?’^66^. Critical realism describes three types of reality: (1) “empirical”: experiences as they are described, (2) “actual”: events that objectively have occurred, and (3) “real”: the mechanisms that contain the forces within a social structure to create an event (Fig. 1) as a way to organize data for context-mechanism-outcome evaluation^45,46,67^. As shown in Fig. 1, critical realist inquiry relies on abduction to move between theory and data categorized in the “empirical” and “actual” realms to identify what must be true for something to occur, thereby arriving at the mechanism^68^. We moved between data categorized in each type of reality to understand which experiences were specific to a participant verses experiences that reflected mechanisms embedded in the wider social structure that applied to the majority of PLHIV in our study setting. In this study we are interested in the forces driving climate change-related impacts on HIV health in this rural setting in Western Kenya.

Throughout our analysis we asked, ‘what are the facilitating factors in order for a theme to be true’ while constantly moving between data categorized as “empirical” and/or “actual.” Facilitating factors were used to identify the forces (“real”) that influenced mechanisms leading to outcomes.

Data were categorized by “strength” of support to reflect the consistency of participant responses for each theme. Strength of support was classified as strong when more than half of participants described the theorized mechanism with little or no divergent experiences; moderate when 20–50% of participants reported it with some variation; and limited when fewer than 20% of participants reported it.

## Results

This study cohort included 40 PLHIV (20 intervention, 20 control) evenly divided by gender of household head, who ranged in age from 18 to 60 years (See Table 1). At baseline, 70% of participants were severely food insecure, and HIV viral load detection was 5% (viral load detection limit, ≤ 1000 copies/ml). People primarily grew grains, pulses, dark leafy greens, and nuts and seeds. All interviews took 1.5–2 h.

All participants described their experiences with climate change-related severe weather in their village, either in the recent past (the past 1–5 years) or currently. These included changes in when it rained, and increased severity and duration of flooding, excessive heat, and droughts. Most participants described the effects of these weather changes as lower farming yields and increased crop losses, as compared to historical and more predictable fluctuations in yields. Almost all participants described at least one of five key pathways : (1) weather-related reductions in agricultural yields and income (strong), (2) increased food insecurity and undernutrition (strong), (3) infrastructure erosion, missed clinic visits, and medication non-adherence (moderate), (4) infections (moderate), and (5) displacement and forced migration (limited). These pathways include the four (pathways 2–5 listed above) previously described in Lieber et al. (2021)^10^, between climate change-related severe weather and impacts on HIV health. In many cases participants explained how pathways were interrelated because one mechanism often impacted multiple pathways. For instance, reduced agricultural income compromised access to treatment and at other times increased food insecurity. In addition, participants explained that multiple mechanisms acted upon pathways simultaneously, for example: compromised access to treatment was caused by reduced income, infrastructure loss, and forced migration, illustrating the multi-faceted role climate change may have on accessing treatment.

**Table 1:** Characteristics of study participants at baseline enrollment in *Shamba Maisha* trial.

Characteristics	Mean (SD) or *n* (%)
*N*	40
Gender	
Male	20
Female	20
Age in years, mean (SD)	43 (9)
Marital Status *N* (%)	
Married	14 (35)
Household size, mean (SD)	5.3 (2.5)
Categorical Food Security score, *n* (%)	
Mildly food insecure	1 (2.5)
Moderately food insecure	11 (27.5)
Severely food insecure	28 (70)
Education, years completed, mean (SD)	3.6 (1.6)
Land size farmed in hectares, mean (SD)	2 (1.3)
Number of households growing the following crop categories, *n* (%)	
Grains	40 (100)
White root, tuber, plantains	16 (40)
Pulses	39 (98)
Dark leafy greens	40 (100)
Nuts and seeds	31 (78)
BMI categories *n* (%)	
Underweight (< 18.5 kg/m^2^)	6 (15)
Normal (18.5–24.9 kg/m^2^)	21 (52.5)
Overweight (25.0–29.9 kg/m^2^)	10 (25)
Obese (≥ 30 kg/m^2^)	3 (7.5)
Viral load detection limit, ≤ 1000 copies/ml, *n* (%)	
Undetectable	38 (95)
Detectable	2 (5)
Wealth index, quintiles, *n* (%)	
Lowest	6 (15)
Second	4 (10)
Third	11 (27.5)
Fourth	10 (25)
Highest	9 (22.5)

**Fig. 2:**
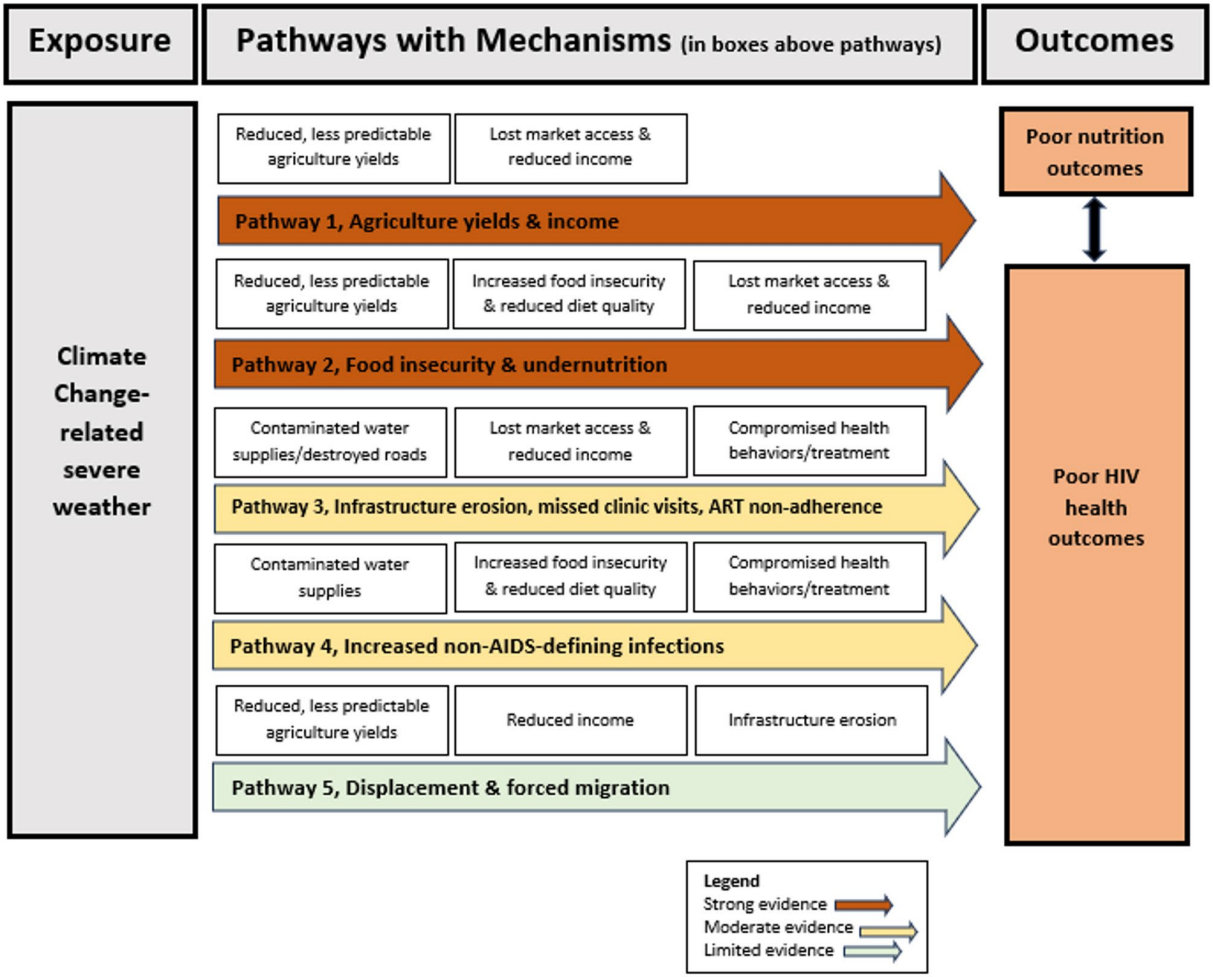
Conceptual framework depicting results of mechanisms and pathways between climate change and impacts on nutrition and HIV health outcomes for farmers living with HIV.

Figure 2 presents a new conceptual framework derived from our results, illustrating these five distinct pathways from exposure of severe weather to poor nutrition and HIV health outcomes for farmers living with HIV. By mapping the interconnected pathways, we show how changes in agricultural yields, food insecurity, infrastructure damage, and displacement operate through specific mechanisms such as reduced income, poor diet quality, and compromised treatment to ultimately impact nutrition and HIV health outcomes. The arrows and evidence grading (strong, moderate, limited) help assess the relative strength of support for each pathway providing a clearer understanding of how climate-related weather shocks cascade through agricultural and health systems.

### Climate change-related reductions in agricultural yields and income (strong evidence)

Climate change impacts on agricultural yields was a dominant pathway and contributed to several other pathways’ impacts on nutrition and HIV health outcomes, either directly or indirectly via reduced income or increased food insecurity. All participants described farming as their primary or only source of household food and income, and most explained that both farm yields and income were greatly reduced in recent years because of climate change-related severe weather. Participants, described farming their land in the past without experiencing the current weather-related crop failures, as illustrated by this farmer’s quote,

*“In the past as we were working*,* we planted cereals in February each year without fail. In January like now everyone would have ploughed their farms. Nowadays we plant in March or April because the patterns of rainfall have changed.”* Man, 52 years.

Participants explained a variety of ways that the climate negatively affected their crops and livestock, such as unpredictable rain, prolonged droughts, damage to soil fertility, and new crops and animal diseases. For example, one woman who experienced repeated farming losses that led to stress over her inability to meet her children’s needs, elaborated on weather changes, and what these changes mean for farming practices.

“*The changes I have seen is that in the past*,* we used to receive so much rain and people would plant by the month of February and crops yields were also quite high. Currently*,* we experience extended dry seasons to the point of people planting in April or March…we also never experienced pest attacks as we currently do…Nowadays floods tend to cause adverse soil erosion leaving infertile*,* dry soil behind. In the past*,* we had trees which slowed down the effects of the floods but since they were cut down*,* floods easily carry away the topsoil and*,* as a result*,* soil fertility has gone down*.” Woman, 58 years.

She went on to compare prior experiences of owning livestock to the present day, that she attributed to changes in weather.

“*I used to have livestock*,* but they are not there anymore because they died from animal diseases…We never used to treat the animals with [veterinary] chemicals*,* yet the animals were just okay… Now*,* they just die. We buy the drugs and call the veterinary doctors who examine the cattle and treat them but still*,* they never do well…nowadays there are more diseases…Or the current weather is full of livestock diseases*.” Woman, 58 years.

Loss of agricultural income impacted participants in multiple, interrelated and reinforcing ways, including inability to purchase food, rebuild destroyed farms, purchase higher-cost climate adaptive seeds, fertilizers, and pesticides, and a lack of funds to pay for transport to clinic. Some participants described how their loss of agricultural income created a vicious cycle of poverty and food insecurity, which they described as hopeless. Others described coping by surviving on less food or removing their children from school as they could no longer pay school fees.

*“You have worked on your farm…like just the other year. But because of the change of weather as a result of climate change*,* you get only three sacks. So that [previous] year that you had ten sacks*,* maybe you consumed six. You sold four for school fees. Now you have gotten only three. Even the one to sell for school fees is not there.”* Man, 31 years.

Participants described yields and income as completely interconnected and the force determining their economic stability. One female participant described how weather shocks that destroyed her crops were linked to a cycle of poverty.

*“It rained too much for two consecutive days with hailstones. All our crops were destroyed…I bought seeds and I lost everything. It [excessive rain} floods the farm thus destroying the crops…Flooding can also make the crops be swept away; hence no harvest and the cycle of poverty continues.”* Woman, 55 years.

A male participant described that, before the current climate-related impacts on yields, he had enough yields to set aside staple crops, such as maize, to feed his family for the coming year, but now climate change left them with nothing and forced them to purchase food.

*“[Normally] when I plant*,* I get enough yields to consume for between six months to one year. Last season my crops were swept by the floods – I had to buy maize from the market*,* even the one we eat in the house. I do not have even vegetables. So*,* I can say the climate change has really dealt our farming a heavy blow…It is connected to climatic change because without rain the crops may not flower. Climate change has also led to increased pests and crop diseases. Again*,* when the rainfall is too much or there is hailstone*,* fruits which haven’t matured are certainly destroyed.”* Man, 42 years.

People described rebuilding their farms or garnering income from non-farming activities, such as, men relocating to cities to earn an income and leaving their wives and children at even greater risk of crop losses due to loss of their farm labor. Although not a dominant theme, one woman said she needed to engage in prostitution to earn money. Some linked climate change to a need to invest in their farm to harvest just enough to eat (Appendix A).

Income losses also made it difficult to purchase the food they would normally consume to diversify their diets and support their HIV health and ART adherence. Participants identified the interconnectedness of climate change-related crop losses, lack of income to purchase agricultural inputs and lack of adequate quantity and quality food as pathways to uncertainty about ART adherence.

*“You know why I can’t get enough money for my food and health? There are several connected reasons. If I wanted to plough my farm and grow rice*,* maize*,* or sorghum*,* I will be worried because floods washed away topsoil…I am not able to hire tractors to work on a larger piece of land and produce sufficient food. I will plough my farm manually…If I would sell my own farm produce and earn at least Kshs. 50*,*000 a season*,* what would prevent me from getting meat at least thrice a week and be healthy? [I would] then take my drugs without so much worry and be strong; and live long. I would gladly ensure strict ART adherence. But that’s not the case. We have so many needs.”* Man, 58 years.

### Climate change-related increased food insecurity and undernutrition (strong evidence)

Losses to food production and lack of non-farming income opportunities combined to perpetuate food insecurity. Increased food prices due to regional climate shocks made dependence on food markets unaffordable especially in the context of reduced income, making food insecurity a strong pathway toward negative impacts on nutrition and HIV health. Participants characterized “the past” as their lives when “we had enough food and a variety of food” and before they noticed “climate change destroying farms and drying up rivers.” Some participants compared their food security from the past to their current situation of food insecurity as “famine” and “starvation” while describing the interconnectedness of rising food prices, and their lack of income (Appendix A).

The below participant’s description illustrates how crop loss from floods led to significant food insecurity and undernutrition:

*“My weight declined so much [after the floods]. I would work so hard*,* the whole day. But I had nothing to eat. The energy spent from my body could not return. If you plough a large portion of land a day*,* you become so tired when you go home in the evening. Then you eat what cannot satisfy your hunger… My health deteriorated so much*,* especially weight… My body shrunk. I was hungry most of the time. I couldn’t sleep at night because of the hunger. Sometimes I could only afford a cup of porridge…Then I would take my drugs and go to bed. I lost a lot of weight.”* Man, 42 years.

His experience reflected a common sequence of interconnected events reported by many participants about the specific vulnerabilities of smallholder farmers living with HIV who need food to take their ART medication and have always relied on their crops for both household food and income to meet their nutrient needs.

Most participants explained that if they were able to produce nutrient-dense vegetables, then they were needed to support their and their children’s health, but they also needed the income from selling vegetables at higher prices when vegetables were scarce. Since this was their only income source, a small number of vegetables would be used for home consumption, while the majority were sold. Income was then used to purchase calorie-dense, micronutrient-poor cereals as a means of survival. Participants universally described this coping mechanism as negatively impacting their HIV health.

*“As a farmer*,* [climate change] has affected my income because there are no vegetables; I would get more money if I were selling vegetables…then I use the money to buy something else; so [now] it’s just vice versa. All is because of weather changes. I can’t diversify my family diet when I get no yield from my farm hence no income because of drought. It’s quite difficult at times such that even if you get some little money*,* instead of buying some beef or eggs or beans for the children*,* I use the money for something else*,* like buy cereals; it is quite difficult to maintain a balanced diet…Moreover*,* considering the eating patterns*,* you are forced to eat the same foods all the time; if it is sardine then it is that sardine over and over again. It’s because during those times*,* there is scarcity of vegetables; I may end up selling almost all my vegetables and remain with just a small amount for the children. I sell almost everything to buy maize to get flour. Drought is very bad for human health.”* Woman, 27 years.

Some people worried about losses of indigenous vegetables commonly grown locally in their diets because they could not grow under the new climactic conditions. Without these vegetables on people’s farms or in the markets, there was concern about future access to these foods.

A minority of participants discussed growing diverse crops in multiple locations which provided access to a variety of foods when floods destroyed some of their plots. However, most participants were limited to one farming location which subjected their food security and diet quality to increased vulnerabilities. Participants asked neighbors or family members for food, but they were often unable to help because they were also suffering from food insecurity. Participants described feelings of shame when asking for food assistance and this deterred seeking help. When asked about food assistance from the government, a few people reported receiving minimal offerings of rice. Some participants noted a link between their children being malnourished and climate change impacting food scarcity and increased prices.

### Medication non-adherence, missed clinic visits, infrastructure erosion (moderate evidence)

Participants consistently reported that their doctor instructed them to take their ART medications with food and at the prescribed times, thus they were motivated to adhere to instructions to improve their health. Study respondents explained that their medications would not be as effective at suppressing HIV, and they would experience severe nausea, weakness, and malaise if they took their medication on an empty stomach. This was a common concern with all participants reporting some degree of food insecurity.

*“One day I was forced to skip [ART medication]. I didn’t get food at all. Now I had to…think properly. I realized that the effect of the drugs was going to be hazardous on an empty stomach. I have told you that I would get dizzy and nauseated if I didn’t get enough food. What about eating nothing completely? I feared for my life*,* so I didn’t take the drugs… This was last year when the floods rendered me destitute.”* Man, 36 years.

Participants missed doses when they lacked sufficient food related to climate-related income losses or low crop productivity. As described by a woman linking her non-adherence to food and water insecurity.

*“I have to eat well and take HIV medication…If I don’t get enough food*,* then I suffer from the side effects of the drugs. When I do not get enough food*,* I lack energy to work on my farm and if I do not work well in my farm then I will not get enough food to help the drugs fight the HIV virus in my body. When I do not get food*,* I contemplate not taking the medication. Moreover*,* when the water is dirty because of weather change I do not feel comfortable swallowing the drugs using it. When weather is bad it affects my income and so I can’t buy the foods I need*,* so when I take the drugs*,* I get severe side effects. Sometimes I would even decide not to take the medication because of lack of food.”* Woman, 56 years.

Entrenched poverty exacerbated by climate change related losses were linked to many respondents reporting missed clinic appointments due to lack of money for transportation. People also reported walking in extreme heat was difficult, especially when undernourished. Washed-out roads due to frequent flooding made transportation more expensive with motorcycle drivers raising their prices.

*“When it rains*,* reaching such places is a challenge since the roads become muddy and impassable. Reaching Minyenya clinic is a hustle [challenge] because the roads are in deplorable condition…The fare is hiked by motorcycle operators. It is very hard to find any means of transport.”* Man, 49 years.

The theme of interconnectedness of climate change-related severe weather impacts on agriculture, food security and income was the explanation all participants gave for their difficulty with adherence or clinic attendance.

*“It’s not been easy for me because there are so many challenges…such as lack of enough food; maybe I need to go to the clinic and yet I haven’t taken any food or maybe you lack transport money. The sun is also shining and destroying crops thus low yields. All these just make someone to get more worried about their HIV status…It’s difficult to walk in the scorching [sun]…it also causes headaches and tiredness. Sometimes when I go to the clinic and spend the whole day there*,* then when I come back home that is when I need to figure out how I will eat and sometimes I do not have money. This sometimes make me feel discouraged from attending clinic*,* but I know I have to go to the clinic to get my HIV medication.”* Woman, 45 years.

Many participants employed adaptation strategies to prevent missing their appointments, such as beginning their journey to the clinic at a certain time of day or calling the clinic to reschedule to avoid the rain. A few people reported their journey to the clinic was delayed due to the weather, only to find upon arrival that the clinic was either closed or they were too late for their appointment (Appendix A).

Participants cited heavy rains and flooding causing them to feel sick while walking to the clinic or to become sick later, yet many still made the journey because this was their only means of receiving their medications. Those who were not able to make their appointment due to flooded roads reported attending the clinic within a few days. One participant believed her children would become orphans if she did not attend the clinic to receive her medication, so she was willing to walk long distances through severe weather to prevent this.

Participants described climate change-related impacts on infrastructure to include washed out roads and bridges, interrupted water delivery and malfunctioning drainage systems, destruction of housing and farms, and to a lesser degree damaged clinics. Flooded roads prevented participants from accessing clinics and markets, which they explained had a direct impact on their HIV health, their income, and ability to purchase food or other necessities. One participant described how flooded roads made it impossible to reach markets to sell her crops.

*“The roads are flooded so I can’t take anything to market to sell so my income is affected.”* Woman, 45 years.

### Climate change-related infections (moderate evidence)

Weather shocks affected infections in several mutually interacting and reinforcing ways. These include compromised immunity due to lack of food; malaria due to stagnant water; waterborne diseases due to poor water quality; and lack of access to health facilities for treatment.

*“Even though we rarely face chronic diseases*,* malaria and flu were common during this recent rainy season. Malaria is more common now due to stagnant pools of water that breed mosquitoes during rains.”* Man, 38 years.

Another participant described the relationship between changes in water and subsequent diarrhea, malaria, and cholera.

*“It [severe weather] brings about different diseases such as diarrhea*,* malaria…among others…Dust brings colds during the sunny season while the rainy seasons brings about cholera infections as floods carry dirty water; cholera also breaks out during the dry season because the little water that is available can be contaminated and be shared across a large population.”* Woman, 58 years.

Participants discussed the natural environment, food insecurity and a lack of health resources relating to infectious diseases and attributed food scarcity and poverty to negative impacts on their general health.

*“Here when it is so dry*,* we become prone to cholera and skin diseases. When you come to the clinic with a skin disease the doctor will advise you to take fruits*,* yet you got nothing even to eat. The vicious cycle continues…When it rains here or in the adjacent highlands*,* mosquitoes breed so much that we become prone to malaria. The water from the highland flow and become stagnant here because this is a plain.”* Man, 38 years.

People were concerned that these negative impacts on their health would interfere with their ability to perform farm labor or would weaken their immune system and compromise their HIV health.

### Climate change-related displacement and forced migration (limited evidence)

In a few cases, participants described their house, farm, crops, and livestock being swept away by floods, which led them to migrate to other areas until the waters subsided. Relocation prevented people from farming thus increasing their food insecurity and loss of income, both of which are a threat to HIV health. People tried to rebuild in the same locations since that was the only land they owned, but in some cases, people permanently relocated to higher land because they feared the floods would continue. This would put people at risk of disruption to care and treatment routines or needing to establish new care pathways.

*“Last year and this year I was much disadvantaged*,* more so this year I had to relocate to a nearby [disaster relief] center. I only came back home after the floods had subsided. The floods destroyed my house. I have not even completed repairing the house. It is so disturbing. I can no longer live comfortably where I used to live because of the floods.”* Man, 42 years.

One participant described a change in flooding activity that forced an entire village to migrate because their homes were no longer habitable.

*“Floods have increased because you find when it rained last–places like Modi (a place just within our village) were so affected to an extent that those who live there were forced to relocate to other places…There were some homes that were destroyed completely.”* Man, 33 years.

Participants from counties with flood plains reported similar destruction to their homes, to the extent that they were forced to relocate and if they returned, they would need to build a new house with the expectation that they would experience flooding again. This type of destruction reinforces the viscous cycles of poverty, food insecurity, malnutrition, and increased infections.

*“There are people whose houses were completely flooded; therefore*,* they had to move to schools*,* churches*,* and market centers to seek shelter. Red Cross came to their aid and helped people redo their houses after the floods subsided. Those who had alternative land were advised to move to higher grounds but those who did not have alternative land were forced to construct their houses at the same locations that are still prone to flooding.”* Woman, 38 years.

## Discussion

This study unpacks how PLHIV in rural Kenya experience and respond to the increasing severity and frequency of droughts and floods, and if and how they perceive these climate shocks affect their HIV health. We examine the interconnection between multiple pathways, to better understand if certain pathways are perpetuating, reinforcing, or exacerbating negative effects of climate shocks on HIV health. Guided by Leiber et al.’s (2021) study, we identified some similar pathways between climate change and HIV health, including increased migration and infectious disease prevalence, infrastructure erosion, and food insecurity, and some new pathways between climate change and HIV health including reduced agriculture yields and income. Our study population differed by including only PLHIV who were farmers, hence our study’s strong evidence of lived experiences of reduced agricultural yields and income.

These findings build upon the Lieber et al. conceptual framework by showing the deeply interconnected nature of the pathways linking climate change and HIV health. Specifically, the analysis reveals that agriculture functions not only as one among four other pathways, but as a central and proximal mechanism influencing most others. The Lieber et al. framework groups agriculture with food and nutrition, while this study disentangles these components, showing that disruptions to agricultural yields often initiate cascading effects on food access, income, caregiving capacity, and ultimately health outcomes. Notably, with the exception of infrastructure erosion—which destabilized agricultural production and markets—agriculture emerged as a key entry point through which climate-related impacts were experienced and transmitted across domains.

Using critical realist theory, we examined these pathways involving multiple and interacting social, environmental, and economic structures that shaped the “real” experiences for PLHIV. We did this by using a model of context-mechanism-outcome to isolate forces found as facilitating factors required for an outcome to occur. We found strong evidence of climate change-related events impacting agriculture productivity, yields, and income thereby trapping PLHIV in a cycle of poverty. We also found strong evidence of climate change-related food insecurity and undernutrition via agriculture pathways including loss of farming income. We found moderate evidence for climate change-related compromised treatment adherence activated through the food insecurity pathway and missed clinic visits activated through the loss of income and infrastructure erosion pathways. Moderate evidence was also identified for increased infections through erosion in infrastructure and increased food insecurity and undernutrition pathways. Eroding infrastructure, displacement, and forced migration were indicated but less robust in our data compared with other pathways.

These results are consistent with the three published studies (at the time of writing) using participant interviews to examine the impacts of climate change on PLHIV in agrarian settings. The earliest study, conducted in Tanzania, including single mothers at risk for or living with HIV, reported important gendered vulnerabilities and the emotional burden of persistent food insecurity^69^. By disaggregating agriculture from nutrition, our study builds on this previous study and adds specificity to the circularity of these pathways, demonstrating how compromised nutrition was perceived to interfere with ART adherence and overall health, thereby diminishing labor inputs and exacerbating already compromised agricultural yields. The second study, from South Africa, examining drought impacts on ART adherence, found that weather shocks interfere with HIV care and disrupt adherence through multiple interrelated and bi-directional pathways including agricultural losses and infrastructure erosion^70^. Consistent with our findings, both studies found that agricultural and income losses were proximal to most other pathways disrupting ART adherence^69,70^. Our findings further demonstrate how climate-related agricultural and income losses cause long-run disruptions to livelihoods. Failed harvests reduce household income and food availability, extending food insecurity in subsequent seasons when depleted food stores and additional climate shocks, such as floods, further reduce agricultural yields and exacerbate cyclical challenges to sustained ART adherence. Prior analyses of the *Shamba Maisha* cohort have shown that climate-related agricultural losses can generate emotional strain, anxiety, and stress among PLHIV^71^. Our findings extend this work by illustrating how these emotional experiences are embedded within broader livelihood disruptions, linking psychological distress with material losses in agriculture, income, and nutrition. Taken together, the two *Shamba Maisha* studies provide a more comprehensive picture of how climate change impacts both the psychological and physical dimensions of vulnerability for PLHIV through interconnected livelihood pathways and further exacerbates vulnerabilities, threatening gains in both HIV care and treatment.

Underlying the negative outcomes reported were trade-offs between using limited income to buy food, pay for transportation to clinic, or purchase farming inputs to recover crop losses. Although it is well established that poverty and food insecurity undermine HIV health outcomes^21,72–76^, our findings demonstrate how increasingly frequent, and severe weather shocks constrain households’ ability to recover from these trade-offs over time. Previous studies have similarly shown that competing demands on limited resources can compromise long-term HIV health^25,77,78^; our analysis highlights how climate change (Fig. 2) intensified these pressures by repeatedly eroding the livelihood resources needed to stabilize food security and treatment adherence. These findings suggest that climate-related livelihood shocks intensify resource allocation trade-offs within households, forcing individuals to balance immediate survival needs against investments necessary to maintain both agricultural recovery and sustained HIV care. Figure 2 synthesizes these interrelated pathways, illustrating how climate-related livelihood disruptions cascade through nutrition and HIV health.

The results presented here contribute to this emerging field by advancing a more integrated, structural account of how climate change affects PLHIV in farming communities. Through a critical realist lens, these findings move beyond documenting vulnerability to analyzing how social, economic, and environmental mechanisms interact to exacerbate existing inequalities. Our work also points to agriculture as a crucial entry point for climate adaptation, HIV care integration, and structural intervention to support PLHIV in climate affected regions. Figure 2 can be used to navigate our results holistically, identifying where interventions might be most needed and effective.

Kenyan HIV policies focus on expanding testing and treatment access^79^, contributing to widespread national progress over the past 20 years including a viral suppression rate of 74%^80^ and a reduction in HIV prevalence by more than 50% to 4.3% nationally^51,81^. However HIV prevalence remains markedly high in the Nyanza Region (17%) where this study took place^51,81^. Our findings suggest some of these gains may be increasingly vulnerable if worsening food insecurity and poverty associated with climate shocks are not addressed.

Although the Kenyan constitution entitles all citizens to food and nutrition security^82^, and national policies exist to support smallholder farmers experiencing climate shocks, evidence of implementation remains limited^17,82^, and more than half of Kenyans living with HIV experience food insecurity^83,84^. Kenya’s devolved agriculture extension system, introduced in 2016 to enable counties to implement locally relevant climate-smart programs^82^, could provide an important platform for interventions similar to *Shamba Maisha*. However, programs must account for the specific vulnerabilities faced by PLHIV, including labor shortages due to illness and time away to address health needs which can intensify the adverse impacts of climate shocks on HIV health^85,86^. NGOs already supporting smallholder farmers experiencing climate shocks may offer additional opportunities to reach these populations^87^, but adaptation efforts will be most effective if they explicitly address how living with HIV compounds the challenges of responding to climate-related livelihood disruptions.

Kenya’s Ministry of Health identifies the need for policies to reduce the spread of infectious disease at the community level and in hospitals^88^. Our findings suggest that sustaining reductions in HIV transmission will require policies that also address climate-related drivers of vulnerability among PLHIV, especially drivers operating through agricultural livelihoods, food insecurity, and income instability. Climate-resilient health systems defined by the World Health Organization as health systems that can anticipate, respond to, cope with, recover from, and adapt to climate-related shocks provide a useful framework for strengthening HIV programs in the face of climate change^89^. Such systems emphasize intersectoral governance, climate-informed decision-making, and stronger integration of climate risk data into HIV programming^90^, approaches that could help address the cascading livelihood disruptions identified in this study. In particular, policies and programs that target climate-related reduced crop productivity, income, and infrastructure erosion may help mitigate the downstream effects on nutrition and HIV health. Future studies should therefore prioritize identifying cross-sectoral strategies that integrate climate-smart, nutrition-sensitive agricultural extension into existing HIV care and support frameworks^84^. Given Kenya’s substantial progress in reducing HIV transmission over the past 20 years alongside increasing climate shocks to this region^52,56,91^, our findings make the case for HIV programming to address current and emerging climate-related threats to treatment adherence and long-term HIV health.

### Strengths

Interviewers were native to Western Kenya and members of smallholder farming families who depend on crop yields for their primary source of food and income. This shared experience reduced the inherent power imbalances between interviewers and participants, and the interviewers’ local knowledge enabled them to ask contextually relevant probing questions. The full participation of the interviewers in data analysis strengthened our understanding of the data and informed our interpretations of its meaning enabling us to triangulate findings. As a qualitative study we were able to examine in-depth pathways and mechanisms. While the results are not generalizable, they do present some key insights which may be relevant to PLHIV who live in rural, climate-affected regions and are dependent upon rain-fed agriculture for their livelihood and household nutrition. The mechanisms identified in this study could be relevant for understanding how climate change may affect food security, livelihoods, and health outcomes in similar smallholder farming populations.

### Limitations

Interviews were conducted in 2018, and additional climate shocks, consistent with those described in our study, have continued to occur in Kenya from 2019 to the present time^92^. As climate change continues to intensify, the specific climatic conditions described by participants may have evolved. However, the aim of this study was not to document a single climate shock but rather to examine the structural pathways through which climate change affects the lived experience and health among PLHIV in rural settings. These pathways, including reliance on rain-fed agriculture, climate-sensitive food production, and limited adaptive resources, remain persistent experiences of rural people in Western Kenya. There is risk of social desirability bias, since participants were part of the *Shamba Maisha* study that tracked their ART adherence and clinic attendance. Responses to questions related to how climate change impacted upon medication adherence or clinic attendance may have been influenced by a participants’ fear that disclosing non-adherence or missed appointments would impact their study participation. We tried to address this risk by assuring participants that their responses would not affect their participation in *Shamba Maisha* in any way, and by including interviewers who were not part of the main *Shamba Maisha* study team. Participants were part of a climate adaptive agriculture intervention, and therefore some of the pathways may have been more difficult to discern among participants who started enjoying benefits from the intervention. However, responses regarding how climate change was impacting HIV through the identified pathways did not differ depending on whether participants were enrolled in the intervention arm because the pathways existed for them regardless of if they had learned new ways of farming to mitigate the effects of the pathways.

## Conclusion

PLHIV face specific vulnerabilities to climate change jeopardizing their agriculture-based livelihoods, food, and nutrition security. This in turn can jeopardize prevention, care, and treatment efforts. The gains in HIV health made over the past 40 years depend upon PLHIV accessing ART medication and healthcare services, while also receiving adequate nutrition necessary to support their HIV health. Policy makers and program leaders addressing climate threats to PLHIV in rural areas should examine regional strengths and weaknesses in agriculture and healthcare structures that PLHIV depend upon and focus on cross-sectoral collaborations. These negative impacts could also affect people in urban settings, particularly as food prices rise, infrastructure is destroyed, and infections increase. The heightened risk of poverty for all PLHIV will exacerbate food insecurity risks during climate related food system disruptions.

## Supplementary Information

Below is the link to the electronic supplementary material.

Supplementary Material 1

## Data Availability

The data supporting the findings of this article are available within the paper. For ethical reasons and following the language of our consent form, which limits access to the data, we are unable to share the underlying qualitative data from this study. This is due to our small, geographically confined study population, all of whom are living with HIV and thus are a vulnerable population at risk of exposure to stigma. Requests regarding the data can be sent to the data access committee which includes study authors Tammy Nicastro, [tammynicastro@gmail.com](mailto: tammynicastro@gmail.com) and Sheri Weiser, sheri.weiser@ucsf.edu.
