# Supplementary material for: Pathways between climate change and HIV health in rural Kenya: a qualitative analysis
Source: Sci Rep. 2026 May 8;16:21138. doi: 10.1038/s41598-026-52085-7 (PMC13342556; doi:10.1038/s41598-026-52085-7)
Supplement: Supplementary file 1 — Supplementary Material 1 [file 41598_2026_52085_MOESM1_ESM.docx]

**Appendix A**

| **Pathway** | **Quote** |
| --- | --- |
| **Climate change-related reductions in agricultural yields and income** | *“The amount of money we use in farming has tremendously increased [since the weather changes].If you do not use money on your farm, you will harvest nothing. In the past you could farm and have plenty of harvest at zero costs. Right now, I must buy fertilizer and seeds. In the past I used no money to farm but I still harvested fairly well. Right now, even for me to get just enough to eat, I must pay the price.”* Man, 37 years |
| **Climate change-related increased food insecurity and undernutrition** | *“I do not have enough food to eat. I came from 70kgs to 40 kgs. Weather had changed for the worse and there was no food. I could not afford food as much as my body needed...There was excessive rainfall and it damaged crops…”* Woman, 56 years |
| **Medication non-adherence, missed clinic visits, infrastructure erosion** | *“Sometimes I plan to come to the clinic on foot, but it is raining, and the river has busted its bank so I can’t get to the clinic. Sometimes I try to come after the rains but where I arrive [the clinic is closed]…I get that their clinic time has ended because they stop at 1pm. It is very challenging. These are things that have made me miss my clinic before.”* Woman, 56 years |
